# The relationship between sexual and gender stigma and suicide attempt and ideation among LGBTQI + populations in Thailand: findings from a national survey

**DOI:** 10.1007/s00127-022-02292-0

**Published:** 2022-05-23

**Authors:** Soroush Moallef, Travis Salway, Nittaya Phanuphak, Katri Kivioja, Suparnee Pongruengphant, Kanna Hayashi

**Affiliations:** 1 United Nations Development Programme, Bangkok, Thailand; 2 British Columbia Centre On Substance Use, St. Paul’s Hospital, Vancouver, BC Canada; 3 Faculty of Health Sciences, Simon Fraser University, Burnaby, BC Canada; 4 British Columbia Centre for Disease Control, Vancouver, BC Canada; 5 Centre for Gender and Sexual Health Equity, Vancouver, BC Canada; 6 Institute of HIV Research and Innovation, Bangkok, Thailand

**Keywords:** LGBT, Minority Stress, Suicidality, Thailand, Gender Identity, Sexual Orientation, Stigma

## Abstract

**Purpose:**

Thailand has one of the highest suicide rates in Southeast Asia; yet, little is known about suicidality among lesbian, gay, bisexual, trans, queer, intersex, and other gender and sexually diverse (LGBTQI +) people living in the region, who may experience elevated risk for suicide. We sought to identify the prevalence of lifetime suicidal attempts and ideation among a nationally recruited sample of LGBTQI + people in Thailand. We further examined the relationship between levels of sexual/gender stigma and suicidal attempt and ideation.

**Methods:**

Data were derived from a national online survey of Thai LGBTQI + individuals between January and March 2018. Multivariable logistic regression was used to examine the relationship between sexual/gender stigma scales, adapting a previously validated instrument, and suicide attempt and ideation.

**Results:**

Among 1,290 LGBTQI + participants, the median age was 27 years. The prevalence of suicide attempt and ideation was 16.8% and 50.7%, respectively. In multivariable analyses, after adjusting for potential confounders, experiences of perceived and enacted sexual/gender stigma were independently and positively associated with suicide attempt (adjusted odds ratio [AOR] = 1.25; 95% confidence interval CI:1.10–1.41 and AOR = 1.31; 95% CI:1.11–1.55, respectively) and ideation (AOR = 1.30; 95% CI:1.17–1.43 and AOR = 1.34; 95% CI:1.14–1.58, respectively).

**Conclusion:**

One-sixth of the sample reported a suicide attempt, while a half reported ever contemplating suicide. Both experiences of perceived and enacted sexual/gender stigma were associated with lifetime suicide attempt and ideation. Multi-level interventions are needed to decrease stigma and in turn suicide among LGBTQ + people in Thailand, including anti-discrimination policies and support for mental health and well-being.

## Introduction

In many settings in the world, sexual and gender minorities (SGM), including lesbian, gay, bisexual, transgender, queer, intersex and other gender and sexually diverse (LGBTQI +) people, have been shown to contend with high levels of violence, stigma and marginalization and thereby elevated risks of suicide compared to their heterosexual and cisgender counterparts [1–10]. However, in Southeast Asia, where nationally representative data also show high levels of negative attitudes and rejection towards lesbian and gay people [11], little attention has been devoted to examine suicidal thoughts and behaviours (STB) among SGM. In particular, the most recent population-level study reported that Thailand has one of the highest suicide rates in the region; yet, SGM-specific suicide rates are unknown, as national data do not collect SGM status [12]. Meanwhile, a growing body of evidence shows that Thai SGM experience well-established risk factors for STB [1–4, 13], including pervasive differential treatment and discrimination across multiple sectors of society (e.g., healthcare, education, workplace, media) [14–18]. To date, one study has estimated the prevalence of STB among adult LGBT populations (*n* = 411) in Thailand and found the lifetime prevalence of suicidal ideation and attempt to be 39% and 13%, respectively [19]. However, the findings of Kittiteerasack and colleagues are limited due to their small sample size. In addition, the study did not examine the impact of perceived stigma on STB, as well as among intersex people who remain largely invisible in SGM research in Thailand and globally.

The minority stress theory posited by Brooks and Meyer attributes health disparities among SGM to be at least partially explained by the unique stressors related to living through hostile and stigmatizing societal conditions [20, 21]. SGM stigma consists of the widespread negative view and devalued status of non-heterosexual identities, beliefs and behaviours (sexual stigma) [22], and non-normative gender identities and expressions (gender stigma) [23]. Previous research has indicated the importance of examining the multi-dimensional nature of stigma [24], including enacted and perceived dimensions of stigma, in relation to STB. Enacted stigma refers to overt expressions of stigma, including acts of violence and discrimination, while perceived stigma (or felt stigma) refers to one’s knowledge of their stigmatized condition and the associated stress with expecting or fearing stigma-related harm [22, 24].

Acknowledging the scarcity of data on sexual stigma and STB among SGM in Thailand, and the need for additional international research discerning associations between particular components of sexual stigma and STB, we conducted a cross-sectional analysis of data from SGM living in Thailand. In 2018, to address the limited research among SGM in Thailand, the United Nations Development Programme (UNDP) launched a national online survey on LGBTQI + people. Drawing from this diverse sample, we sought to identify the prevalence of suicide attempt and ideation, as well as the relationships between suicide ideation and attempt and multiple dimensions of sexual and gender stigma (i.e. perceived and enacted stigma). In addition, we sought to examine the effect of social support on the relationship between perceived and enacted dimensions of stigma and STB, as international research has demonstrated the pivotal and mediating role of social support on STB [25–27]. Our hypotheses are that both dimensions of stigma will be associated with both suicidal ideation and attempt and that the level of social support will modify the impact of stigma on suicidal ideation and attempt.

## Methods

### Study design and recruitment of participants

The UNDP’s national online survey was administered between January and March 2018. Several local, regional and national LGBT community organizations from three regions of Thailand, including north (Chiang Mai and Phitsanulok), northeast (Ubon Ratchathani), central (Bangkok), and south (Pattani) provinces, collaborated to recruit study participants through a chain-referral sampling method [28]. Over 24 LGBT-related community organizations were sent survey promotional images to share online and within their social media networks. These organizations acted as initial seeds to recruit the participants. To be eligible to participate in the anonymous online survey, individuals must have provided informed consent, been at least 18 years old, currently reside in Thailand, self-identify as a SGM and have the ability to read and speak the Thai language [28]. Participants could enter a lucky draw upon completion of the survey to win a redeemable gift card. In total, ten survey respondents were randomly selected to win a gift card prize in the amount of ฿1000–฿5000 (Thai Baht; approximately US$30–150). Detailed descriptions of the survey have been published online by UNDP [28]. All LGBTQI + participants who completed the online questionnaire were included in the present analysis. This survey research project was approved by the Asian Institute of Technology Research Ethics Review Committee.

## Study measures

In the present analysis, we had two primary outcomes of interest: lifetime suicide attempt and ideation (yes vs no, respectively). In the survey, participants were asked: “Have you ever attempted suicide?” and “Have you ever contemplated suicide?” Responses included: “never”, “sometimes”, “often”, and “prefer not to disclose”. Individuals who responded often or sometimes were categorized as “yes” and individuals who responded never were categorized as “no”. We excluded individuals who preferred not to disclose these primary outcomes from the analysis (*n* = 60).

The main explanatory variables of interest included two interval measures of stigma: perceived and enacted stigma. A previously validated 12-item sexual stigma scale was adapted and used [24, 28], which included a 5-item measure in the perceived stigma sub-scale and a 7-item measure in the enacted stigma sub-scale. The sexual stigma scale was adapted to the Thai context to include gender stigma through collaborations between UNDP and LGBT civil society organizations [28]. The questions are shown in Table 1. The term “LGBT” was translated as “people of diverse gender” in Thai, which can refer to all SGM as biological sex, gender, and sexuality are widely conflated in Thai language [25, 27, 28]. This differs from the Western context, where distinctions are made between biological sex, gender, and sexuality [28]. Both sub-scales were lifetime measurements and items were scored on a Likert scale ranging from 1 (‘never’) to 7 (‘always’). The perceived and enacted stigma sub-scales demonstrated acceptable internal consistency (Cronbach’s α = 0.70 and α = 0.73, respectively).

**Table 1:** Questions used to assess perceived and enacted stigma among sexual and gender minorities in Thailand

	Question
Perceived stigma	How often have you heard that LGBT are not normal?
	How often have you had to pretend that you are straight to be accepted?
	How often have you heard that LGBT grow old alone?
	How often have you felt your family was hurt and embarrassed because you are LGBT?
	How often have you felt you had to stop associating with your family because you are LGBT?
Enacted stigma	How often have you lost your straight friends because you are LGBT?
	How often have you been made fun of or called names for being LGBT?
	How often have you lost a place to live for being LGBT?
	How often have you lost a job or career opportunity for being LGBT?
	How often have you been harassed by the police for being LGBT?
	How often have you been hit or beaten up for being LGBT?
	How often have you been sexually assaulted for being LGBT?

A previously validated 12-item sexual stigma scale was adapted and used [24, 28], which included a five-item measure in the perceived stigma sub-scale and a seven-item measure in the enacted stigma sub-scale. Items were scored on a Likert scale ranging from 1 (‘never’) to 7 (‘always’). The sexual stigma scale was adapted to the Thai context to include gender stigma through collaborations between UNDP and LGBT civil society organizations [28]

**Table 2:** Characteristics and prevalence of perceived/enacted stigma and suicide attempt/ideation among lesbian, gay, bisexual, transgender, genderqueer, intersex and other gender identity people in Thailand (*n* = 1290)

Variable	Perceived stigma^a^ (%)		Enacted stigma^a^ (%)	
	Lower (< 3.8) (*n* = 599, 46.4)	Higher (> = 3.8) (*n* = 691, 53.6)	Lower (< 1.6) (*n* = 676, 52.4)	Higher(> = 1.6) (*n* = 614, 47.6)
Suicidal ideation	237 (39.6)	417 (60.3)	291 (43.0)	363 (59.1)
Suicidal attempt	61 (10.2)	156 (22.6)	82 (12.1)	135 (22.0)
Age (median, IQR)	27 (23–32)	27 (23–33)	27 (23–33)	27 (24–33)
Bachelor’s degree or higher education	464 (77.5)	531 (76.8)	553 (81.8)	442 (72.0)
HIV positive	19 (3.2)	15 (2.2)	11 (1.6)	23 (3.7)
Ever received HIV testing	231 (38.6)	304 (44.0)	217 (32.1)	318 (51.8)
Place of residence and birth (discordant vs. concordant)	427 (71.3)	492 (71.2)	498 (73.7)	421 (68.6)
Health insurance scheme				
Universal coverage	273 (45.6)	316 (45.7)	310 (45.9)	279 (45.4)
Public/state/government	72 (12.0)	86 (12.4)	86 (12.7)	72 (11.7)
Social security	254 (42.4)	289 (41.8)	280 (41.4)	263 (42.8)
Monthly income in Thai baht (≤ ฿30,000)	484 (80.8)	556 (80.5)	534 (79.0)	506 (82.4)
Unemployed (vs. employed/student)	33 (5.5)	41 (5.9)	33 (4.9)	41 (6.7)
Difficulty accessing routine healthcare services	62 (10.4)	99 (14.3)	68 (10.1)	93 (15.1)
Difficulty accessing mental healthcare services	76 (12.7)	179 (25.9)	98 (14.5)	157 (25.6)
Social support^b^ (median, IQR)	5.3 (4.3–6.1)	4.5 (3.5–5.6)	5.0 (4.1–6.0)	4.7 (3.6–5.6)
Gay men	84 (14.0)	128 (18.5)	75 (11.1)	137 (22.3)
Lesbian women	112 (18.7)	110 (15.9)	168 (24.9)	54 (8.8)
Transmen	108 (18.0)	95 (13.7)	124 (18.3)	79 (12.9)
Transwomen	81 (13.5)	124 (17.9)	40 (5.9)	166 (27.0)
Genderqueer/non-binary	70 (11.7)	97 (14.0)	86 (12.7)	81 (13.2)
Bisexual men/women	55 (9.2)	59 (8.5)	83 (12.3)	31 (5.0)
Intersex	32 (5.3)	39 (5.6)	37 (5.5)	34 (5.5)
Other sexual or gender minority	56 (9.3)	39 (5.6)	63 (9.3)	32 (5.2)
Biological sex (female vs. male)	379 (63.3)	350 (50.7)	526 (77.8)	203 (33.1)

*IQR* interquartile range, *CI* confidence interval

^a^Higher = greater than or equal to the median perceived/enacted stigma score; Lower = less than the median perceived/enacted stigma score

^b^Multidimensional Scale of Perceived Social Support (MSPSS), responses range from 1 to 7 (strongly disagree to strongly agree)

We assessed gender identity utilizing the “two-step” approach recommended as best practice by the Gender Identity in U.S. Surveillance and the Williams Institute at the University of California, Los Angeles School of Law [32]. We asked participants: “What is your birth sex according to your ID card?” (response options included: “male or female”) and “What is your deeply felt sense of gender identity?” Response options included local terms to facilitate accurate self-identification: “male/man, female/woman, *tom*/*ponae* (a masculine lesbian woman), *sao praphet song* (transgender woman), *kathoey* (transgender woman, a word used mainly by those who identify as kathoey), genderqueer/gender non-conforming, other”. Sexual attraction was assessed by a third question: “Who are you attracted to?” Responses included: “males only, females only, both males and females, only transmen/*tom*, only transwomen/*kathoey*/*sao praphet song*, people of all genders, not sexually attracted to anyone, don’t know”. From these three questions, eight subgroups were created, which included: lesbian woman, defined as a cis (i.e., gender identity corresponds with biological sex) woman attracted to women or self-identified as lesbian in question #2 (“What is your deeply felt sense of gender identity?”); gay man, defined as a cis man attracted to men or self-identified as gay in question #2; bisexual man, defined as a cis man attracted to both men and women; bisexual woman, defined as a cis woman attracted to both men and women; transgender man, defined as assigned female at birth and identified as male, *tom* or *ponae* (individuals who identify as *tom* or *ponae* are included into the transgender man category as the terms can refer to either trans man or masculine presenting lesbian) [28]; transgender woman, defined as assigned male at birth and self-identified as female, *sao praphet song* or *kathoey*; genderqueer/non-binary, defined as assigned male or female at birth and self-identified as non-binary regardless of sexual attraction; and other, which includes respondents who did not fit in any of these categories. The categories of bisexual men (*n* = 21) and women (*n* = 93) were combined after observing low frequencies in the bisexual men category. A ninth subgroup was created by asking participants: “Were you born with a variation of sex characteristics (this is sometimes called intersex)?” Individuals who responded “Yes” were categorized as intersex regardless of their reported gender identity or sexual attraction due to the low number of respondents self-identifying as intersex.

A range of socio-demographic variables were included as secondary explanatory variables, including: age (continuous); education (≥ bachelor’s degree vs. ≤ Por Wor Sor, Por Wor Tor or diploma); place of residence and birth (discordant vs concordant), defined as whether the participant’s current place of residence is the same as their place of birth; monthly income (≤ ฿30,000 vs. > ฿30,000 in Thai Baht; approximately US$930); employment status (unemployed vs. employed or student); and perceived social support, assessed using the Multidimensional Scale of Perceived Social Support (MSPSS) [33], which includes 12 items divided into three four-item scales to assess social support among family, friends, and significant others [33]. Responses are scored on a seven-point Likert scale (1 = strongly disagree to 7 = strongly agree) [33]. We examined the Cronbach’s alpha for the MSPSS and found excellent internal consistency and reliability (Cronbach’s α = 0.91). A range of healthcare-related characteristics were also included: ever received HIV testing (yes vs. no); type of health insurance scheme, defined as the type of healthcare coverage either the Civil Servant Medical Benefit Scheme (CSMBS, for government employees, their families and retirees), the Social Security Scheme (SSS, for individuals in formal employment), or Universal Coverage Scheme (UCS, those not eligible for CSMBS or SSS, which is approximately 75% of the population) [34] (CSMBS vs. SSS vs. UCS [reference]); and self-reported difficulty accessing routine healthcare services (difficult vs moderate/easy); self-reported difficulty accessing mental healthcare services (difficult vs. moderate/easy).

## Data analyses

Bivariable logistic regression was used to estimate the crude relationships between the explanatory variables of interest and both outcome measures. Two multivariable models were constructed using the explanatory variables of interest and each of the outcome variables (suicide ideation and attempt), adjusted for all socio-demographic variables as well as subgroups of LGBTQI + that were associated in bivariable analyses at the *p* < 0.05 level (due to sample size concerns). In the sub-analysis, we explored whether perceived social support might modify the effect of SGM stigma on suicide attempt and ideation by examining the statistical significance of an interaction term (*p* < 0.05). According to UNDP in Thailand, transgender and intersex people (who are often viewed as transgender in the Thai context) have been documented to experience particularly high levels of hostility and stigma due to strict societal expectations for individuals to conform to gender norms [28]. We thus explored the within-group differences in the mean SGM stigma and social support scores among cisgender (those whose gender identity corresponds with their assigned biological sex) and transgender/intersex (including transmen, transwomen, and intersex) participants. We compared the mean scores between cisgender and transgender/intersex groups using the Mann–Whitney *U* (Wilcoxon) test. All *p* values were two sided and all statistical analyses were conducted using R, version 3.6.0 [35].

## Results

In total, the analytic sample included 1290 LGBTQI + participants (Table 2), including 212 (16.4%) gay men, 222 (17.2%) lesbian women, 203 (15.7%) transgender men, 206 (16.0%), transgender women, 167 (12.9%) genderqueer/non-binary persons, 114 (8.8%) bisexual men/women, 71 (5.5%) intersex persons and 95 (7.4%) people with other gender identities or sexual orientations. The median age was 27 years (Quartile [Q] 1, 3: 23, 33), with the majority of the sample employed or being a student (1276, 94.5%) and with a bachelor or more advanced degree (995, 77.1%). Most participants resided in the Greater Bangkok (730, 56.6%) region, followed by Central Thailand (165, 12.8%), North East Thailand (156, 12.1%), North Thailand (155, 12.0%), and South Thailand (78, 6.0%) region (data not shown). The distribution of perceived stigma scores across the sample was approximately normally distributed (mean: 3.9, standard deviation: 1.8; median: 3.8, Q 1, 3: 2.8–4.8), while the enacted stigma scores were skewed to the right (mean: 1.8, standard deviation = 0.9; median: 1.6, Q 1, 3: 1.1–2.3). Overall, the prevalence of lifetime suicide attempt and ideation was 16.8% (95% CI: 14.8–19.0) and 50.7% (95% CI: 47.9–53.4), respectively. The prevalence of suicide ideation and attempt was similar across sub-groups of LGBTQI + participants, except for genderqueer/non-binary people and transgender women who reported higher lifetime prevalence of suicide attempt (27 and 21%, respectively).

The first multivariable model using suicide attempt as the outcome is shown in Table 3. As shown, both higher levels of perceived stigma (adjusted odds ratio [AOR] = 1.25; 95% confidence interval [CI]: 1.10–1.41) and enacted stigma (AOR = 1.31; 95% CI: 1.11–1.55) were independently and positively associated with lifetime suicide attempt. Social support was also independently and negatively associated with lifetime suicide attempt (AOR = 0.79; 95% CI: 0.70–0.89).

**Table 3:** Multivariable logistic regression analyses of the relationship between perceived/enacted stigma and suicidal attempt among LGBTQI + people in Thailand (*n* = 1290)

	Unadjusted odds ratio (95% CI)	*P* value	Adjusted odds ratio (95% CI)	*P* value
Perceived stigma^a^	1.43 (1.29–1.60)	< 0.001	1.25 (1.10–1.41)	0.001
Enacted stigma^a^	1.62 (1.40–1.88)	< 0.001	1.31 (1.11–1.55)	0.002
Age^a^	0.98 (0.96–1.00)	0.044	0.99 (0.96–1.01)	0.298
Education^b^	0.59 (0.43–0.82)	0.001	0.80 (0.56–1.15)	0.220
Ever received HIV testing	2.80 (1.32–5.65)	0.005	1.30 (0.93–1.80)	0.121
Place of residence and birth (discordant vs. concordant)	0.85 (0.60–1.18)	0.332	0.88 (0.62–1.25)	0.490
Health insurance scheme				
Universal (gold card)	Reference	–	Reference	–
Public/state/government	0.82 (0.51–1.29)	0.406	1.02 (0.62–1.66)	0.929
Social security	0.61 (0.44–0.84)	0.002	0.72 (0.51–1.03)	0.074
Monthly income (≤ ฿30,000 vs. > ฿30,000)	2.04 (1.33–3.24)	0.002	1.64 (1.01–2.77)	0.054
Unemployed (vs. employed/student)	1.78 (1.01–3.01)	0.038	1.25 (0.67–2.23)	0.469
Difficulty accessing routine healthcare services	1.21 (0.78–1.82)	0.378	0.96 (0.58–1.54)	0.867
Difficulty accessing mental healthcare services	1.35 (0.95–1.90)	0.090	1.00 (0.67–1.49)	0.988
Social support ^a,c^	0.71 (0.64–0.79)	< 0.001	0.79 (0.70–0.89)	< 0.001
Gay men^d^	0.93 (0.62–1.38)	0.739	–	–
Lesbian women^d^	0.84 (0.55–1.24)	0.392	–	–
Transmen^d^	0.69 (0.44–1.05)	0.097	–	–
Transwomen^d^	1.38 (0.94–1.99)	0.091	–	–
Genderqueer/non-binary^d^	1.37 (0.90–2.04)	0.127	–	–
Bisexual men/women^d^	0.86 (0.48–1.43)	0.568	–	–
Intersex^d^	0.80 (0.38–1.52)	0.527	–	–
Other sexual or gender minority^d^	1.26 (0.73–2.09)	0.390	–	–

Covariates were selected based on a conceptual model identifying potential confounders that could theoretically influence the relationship between SGM stigma and attempted suicide. Covariates related to sexual and gender identity were not associated at the *p* < 0.05 and therefore not included in the multivariable model.

*CI* confidence interval

^a^Per score/year increase

^b^ ≥ Bachelor’s degree vs. ≤ Por Wor Sor, Por Wor Tor, or diploma

^c^Multidimensional Scale of Perceived Social Support (MSPSS), responses range from 1 to 7 (strongly disagree to strongly agree)

^d^vs. all other categories of LGBTQI + participants

The second multivariable model using suicide ideation as the outcome is shown in Table 4 and mirrors the same trends as the first multivariable model. Specifically, among the sample, higher levels of perceived (AOR = 1.30; 95% CI: 1.17–1.43) and enacted (AOR = 1.34; 95% CI: 1.14–1.58) SGM stigma were independently and positively associated with suicidal ideation. We also observed independent and negative associations between higher levels of social support (AOR = 0.72; 95% CI: 0.65–0.79) and SSS (AOR = 0.63; 95% CI: 0.48–0.82) and CSMBS (AOR = 0.64; 95% CI: 0.43–0.95) health insurance schemes and lifetime suicide ideation. An independent and positive association was observed between monthly income (≤ ฿30,000) and lifetime suicide ideation (AOR = 1.57; 95% CI: 1.11–2.23).

**Table 4:** Multivariable logistic regression analyses of the relationship between perceived/enacted stigma and suicidal ideation among LGBTQI + people in Thailand (*n* = 1290)

	Unadjusted odds ratio (95% CI)	*P* value	Adjusted odds ratio (95% CI)	*P* value
Perceived stigma^a^	1.47 (1.35–1.60)	< 0.001	1.30 (1.17–1.43)	< 0.001
Enacted stigma^a^	1.69 (1.48–1.96)	< 0.001	1.34 (1.14–1.58)	0.001
Age^a^	0.96 (0.94–0.97)	< 0.001	0.96 (0.94–0.98)	0.001
Education^b^	0.57 (0.44–0.74)	< 0.001	0.87 (0.64–1.18)	0.363
Ever received HIV testing	1.59 (0.80–3.29)	0.194	1.08 (0.83–1.40)	0.570
Place of residence and birth (discordant vs. concordant)	0.95 (0.76–1.18)	0.632	1.14 (0.87–1.50)	0.348
Health insurance scheme:	1.01 (0.79–1.29)	0.935	–	–
Universal (gold card)	Reference	–	Reference	–
Public/state/government	0.53 (0.37–0.75)	< 0.001	0.64 (0.43–0.95)	0.027
Social security	0.52 (0.41–0.66)	< 0.001	0.63 (0.48–0.82)	0.001
Monthly income (≤ ฿30,000 vs. > ฿30,000)	2.30 (1.73–3.07)	< 0.001	1.57 (1.11–2.23)	0.011
Unemployed (vs. employed/student)	1.55 (0.96–2.52)	0.075	1.03 (0.60–1.76)	0.921
Difficulty accessing routine healthcare services	1.51 (1.08–2.12)	0.016	1.19 (0.81–1.77)	0.383
Difficulty accessing mental healthcare services	1.67 (1.26–2.21)	< 0.001	1.22 (0.87–1.70)	0.245
Social support^a,c^	0.65 (0.59–0.71)	< 0.001	0.72 (0.65–0.79)	< 0.001
Gay men^d^	1.03 (0.77–1.39)	0.819	–	–
Lesbian women^d^	0.87 (0.65–1.16)	0.334	–	–
Transmen^d^	0.77 (0.57–1.05)	0.096	–	–
Transwomen^d^	1.01 (0.75–1.37)	0.932	–	–
Genderqueer/non-binary^d^	1.26 (0.91–1.75)	0.167	–	–
Bisexual men/women^d^	1.38 (0.93–2.04)	0.109	–	–
Intersex^d^	0.74 (0.46–1.20)	0.224	–	–
Other sexual or gender minority^d^	1.19 (0.78–1.82)	0.414	–	–

Covariates were selected based on a conceptual model identifying potential confounders that could theoretically influence the relationship between SGM stigma and attempted suicide. Covariates related to sexual and gender identity were not associated at the *p* < 0.05 and therefore not included in the multivariable model

*CI* confidence interval

^a^Per score/year increase

^b^ ≥ Bachelor’s degree vs ≤ Por Wor Sor, Por Wor Tor, or diploma

^c^Multidimensional Scale of Perceived Social Support (MSPSS), responses range from 1 to 7 (strongly disagree to strongly agree)

^d^vs. all other categories of LGBTQI + participants

**Fig. 1:**
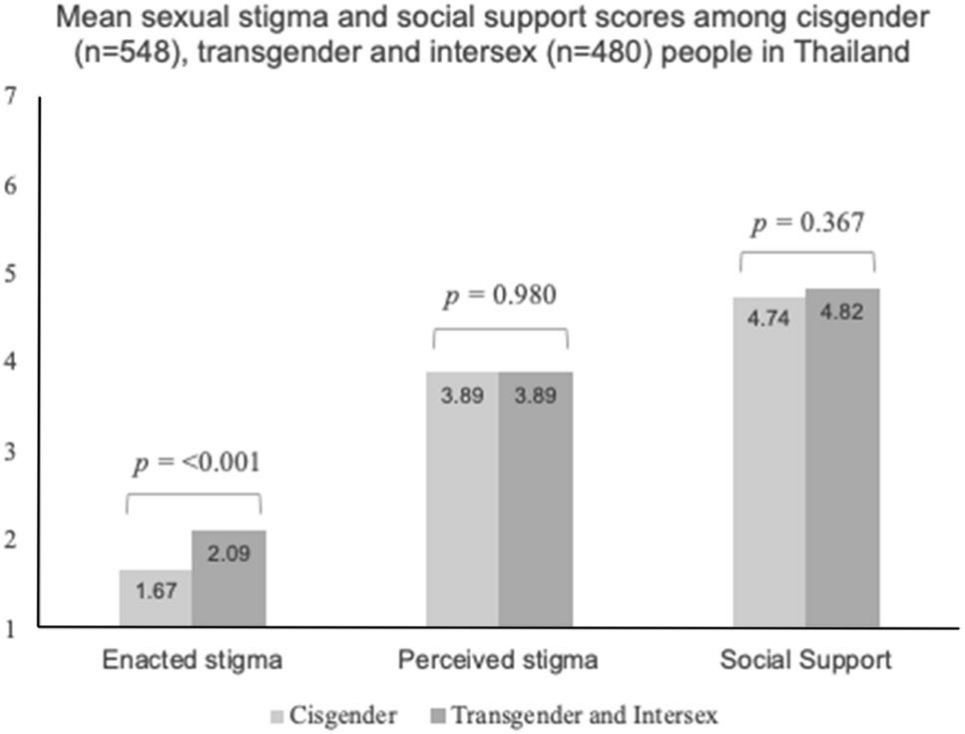
Mean SGM stigma and social support scores among cisgender and transgender/intersex people in Thailand (*n* = 1028). Mann–Whitney *U* (Wilcoxon) tests were used to compare scores between groups. All *p* values were two sided. The enacted and perceived stigma scores ranged from 1 (“never”) to 7 (“always”). Similarly, the social support scores ranged from 1 (“strongly disagree”) to 7 (“strongly agree”)

In the sub-analysis, we observed no significant interaction effect of social support on the relationship between enacted stigma and suicide attempt (*p* = 0.215) and ideation (*p* = 0.839). Similarly, we observed no significant interaction effect of social support on the relationship between perceived stigma and suicide attempt (*p* = 0.322) and ideation (*p* = 0.711). The mean social support and SGM stigma scores among cisgender (*n* = 548) and transgender/intersex participants (*n* = 480) are shown in Fig. 1. Among cisgender participants, the mean scores of perceived and enacted stigma and social support were: 3.89, 1.67, and 4.74, respectively. The mean scores among transgender and intersex participants were: 3.89, 2.09, and 4.82, respectively. Transgender and intersex participants had higher levels of enacted stigma (*p* < 0.001) compared to the cisgender counterparts.

## Discussion

Among our nationally recruited sample of LGBTQI + people in Thailand, approximately 17% reported a suicide attempt in their lifetime, while 51% had ever contemplated suicide. These rates were similar across subgroups of LGBTQI + participants, except for genderqueer/non-binary and transgender women who reported higher lifetime prevalence of suicide attempt. In the multivariable analyses, perceived and enacted stigma were both independently and positively associated with both suicide attempt and ideation. In the sub-analyses, the level of perceived stigma and social support were similar between transgender, intersex and cisgender participants. However, transgender and intersex participants reported higher levels of enacted stigma compared to their cisgender counterparts.

The lifetime prevalence of suicide ideation found in our study was greater than the prevalence estimates from another community-based sample of LGBT in Thailand. Specifically, Kittiteerasack and colleagues (2018) estimated the lifetime prevalence of suicide attempt and ideation among LGBT people (*n* = 411) to be 13% and 39%, respectively [19]. It is important to note that our sample characteristics were markedly different in some respects, including a majority of their sample reporting biological male sex (90.5%) and cisgender (76.6%), while our sample had a majority reporting biological female sex (56.5%) and a minority reporting being cisgender (42.5%). We could not locate other comparable estimates of STB among SGM in Thailand, highlighting the need for further research on STB in the Thai context. However, a systematic review and meta-analysis of STB among sexual minority adults living in North America, Western Europe, Australia, and New Zealand, reported a lifetime prevalence of suicide attempt of 16% [36]. The high prevalence of STB in our study is a cause for concern and warrants additional suicide prevention efforts for SGM. This could include augmenting and integrating suicide prevention efforts into existing health programmes (such as HIV and sexual health services), such as the Key Population-led Health Services (KPLHS) model developed in Thailand in 2015 by and for SGM and other priority populations (e.g., sex worker populations) [37, 38].

The KPLHS model has demonstrated effectiveness in task shifting the service delivery of HIV and sexual healthcare to trained lay providers who are members of the key population [37, 38]. The KPLHS model is guided by three principles, including: (1) non-judgmental, affirming, and confidential care (“KP-friendliness”); (2) accessible care, defined as low or no cost and geographically accessible; and (3) quality care that adheres to national standards of best practices in healthcare delivery [37]. This model has been successful in delivering systematic training and certification to lay providers in providing point-of-care HIV/STI testing, uptake of pre- and post-exposure prophylaxis (PrEP/PEP), as well as treatment service linkages, and individual case management [37]. Augmenting and integrating suicide prevention efforts in KPLHS could be an effective strategy in this setting and should be considered along with expanding culturally-sensitive mental healthcare services for SGM in Thailand [39], including SGM-specific counselling services [14].

Our findings also show that both perceived and enacted stigma were significantly associated with suicide attempt and ideation. These findings are in line with the minority stress model [20] and the international literature [1–6]. In addition, Kittiteerasack and colleagues also found that both general psychological factors (e.g., stress and loneliness measures) and minority stress-related factors (e.g., discrimination, victimization, internalized homophobia) were associated with indicators of suicide risk among LGBT in Thailand [19]. Taken together with our findings, and considering the harmful impacts of stigma on health [22, 37], it is clear that mitigating SGM stigma in society is integral to achieving suicide prevention, as well as to improve the health and well-being of SGM. Our﻿ findings support the previous calls for multi-level interventions to address SGM stigma and suicide among SGM in a holistic manner [15, 25, 38]. These includes structural interventions (i.e., public policies) that aim to protect SGM from the impacts of stigma, such as anti-discrimination policies in healthcare, education, and community settings [28, 41].

A recent review on the social inclusion of SGM in Thailand has identified that the socio-legal context in Thailand poses numerous participatory barriers for SGM that have been described as a failure to acknowledge the existence of SGM in Thai society [41]. For instance, change of gender is not legally recognized in Thailand, which creates barriers particularly for transgender people who must contend with incorrect government-issued identity cards and be continuously subjected to degrading processes to prove their identities [41]. This barrier has been shown to create severe issues and precarious encounters in employment, foreign travel, education, and access to healthcare [41]. In addition, legal marriage in Thailand is only recognized between people of the opposite sex, which excludes SGM from establishing families, receiving marital benefits (e.g., pensions, tax benefits), and partaking in important decision-making for their partners (e.g., hospital visitation, medical decision making) [41]. The strongest priorities identified in the review to mitigate the ongoing marginalization of SGM in Thai society, and in turn reduce the levels of SGM stigma, included: general SGM anti-discrimination laws, legal recognition of gender identity and marriage [41]. Research in the USA on the effect of the social and legal climate (i.e., anti-discrimination and same-sex marriage laws) on the health of sexual minorities supports these priorities [42–44]. In particular, states with protective policies for sexual minorities were significantly protective of known risk factors for STB (e.g., generalized anxiety disorder, dysthymia, among other psychiatric disorders) compared to states without these policies [44].

Of concern, in 2018, a national survey (*n* = 861) of non-LGBT people’s perceptions and attitudes towards SGM in Thailand showed that most respondents did not support equal rights for SGM, including 52.9% who did not agree with legal change of gender and only 46.6% who were supportive of same-sex marriage [28]. It is conceivable that the continued differential treatment of SGM in the legal-political context may serve to legitimize and perpetuate stigma against this population especially among those who do not view SGM as deserving of equal rights. SGM are entitled to equal access to the same institutions and benefits that their heterosexual citizens receive. Our findings strengthen the call for equal access for protections for the safety and safe expression of SGM in the political landscape of Thai society especially given the beneficial effects of these types of legal-political changes on the health of sexual minorities seen in the USA [42–44]. Individual-level support is also needed to ensure that SGM have access to culturally sensitive mental healthcare to address the harmful impacts of SGM stigma and reduce STB risk [14].

In the sub-analysis, compared to cisgender counterparts, transgender and intersex people reported higher levels of enacted stigma despite having almost identical levels of perceived stigma and social support. The higher levels of violent forms of stigma against transgender and intersex people is consistent with Thai research on these communities [6, 18, 25, 47]. In addition, although transgender communities share many of the same predictors of STB as other LGB populations, there are also distinct predictors for STB among these communities [6], including gender dysphoria and difficulties in access to gender affirming care [6]. Intersex individuals may share some similar challenges as other SGM people [8]; however, little research has examined these challenges in relation to STB [9]. Indeed, intersex people are one of the least represented SGM in the literature, warranting further research among this population [8]. We also did not observe significant associations between subgroups of LGBTQI + participants and STB among our sample, which is aligned with the findings of Kittiteerasack and colleagues [19]. Internationally, research among SGM tends to show that bisexual and transgender individuals are at increased risk for STB [6, 7, 48, 49]. Hence, future research should explore differences among subpopulations of SGM in Thailand, and there is a need to tailor public health interventions to the diverse needs of different SGM populations. [12].

Thailand has one of the highest suicide rates in Southeast Asia with considerable differences seen in the crude suicide rates between males and females, specifically in 2019 the rate was five times higher among males (15.0 per 100,000 population) than females (2.9 per 100,000 population) [12]. National data unfortunately does not collect information on SGM status, which makes it difficult to determine the burden of suicide among SGM nationally. There is therefore a need for national data and government agencies to assess and disaggregate national rates by SGM status to support suicide prevention efforts among Thai SGM.

Our study has several limitations. First, our method of chain-referral sampling likely introduced some selection bias. Specifically, a systematic review has shown that sampling from LGB community venues in predominantly Western contexts tends to overrepresent sexual minorities who are employed, have higher educational attainment, and who have a history of suicidal ideation [50]. If these biases similarly affected our sample, we may have overestimated the prevalence of suicidality in the population. As recommended by the systematic review [50], further research that adjusts for frequency of venue attendance is needed, especially to understand the impact of this sampling strategy on estimates of stigma and STB among Thai SGM. Second, our self-reported measures are limited by reporting bias and the suicide attempt measure is likely affected by survival bias. Additionally, our assessment of SGM stigma does not include measures related to internalized stigma (another dimension of stigma) [22]. Of note, our social support measurement quantifies the average level of support received from a significant other, friends, and family. This limits our ability to discern the impact of different types of social supports on the risk of STB among Thai SGM and may have resulted in finding no significant effect modification by the social support variable in our study. Future research in the Thai context should aim to examine the effect between these types of social support given the importance of each type of social support, as seen in international contexts [27, 48, 49].

## Conclusion

Among our nationally recruited sample of LGBTQI + people, the lifetime prevalence of suicide attempt and ideation was 16.8% and 50.7%, respectively. In addition, individuals who reported higher levels of perceived and enacted stigma were significantly more likely to report lifetime suicide ideation and attempt. Further, transgender and intersex individuals were found to experience higher levels of enacted stigma than their cisgender counterparts. There is a need for further research on the burden of STB among SGM in Thailand and multi-level interventions are needed to address SGM stigma and suicide among SGM in Thailand. These interventions should include: increasing suicide prevention efforts, the adoption of anti-discrimination policies across multiple sectors of society, and provisions of culturally competent mental healthcare for SGM. 

## Data Availability

The data and code used in this study are available upon request and approval from UNDP.
